# Prefrontal electrical stimulation in non-depressed reduces levels of reported negative affects from daily stressors

**DOI:** 10.3389/fnhum.2017.00063

**Published:** 2017-02-14

**Authors:** Nick J. Davis

**Affiliations:** Department of Psychology, Manchester Metropolitan UniversityManchester, UK

**Keywords:** tDCS, TMS, neuroethics, safety, cognition

## Abstract

Advances in neuroscience and pharmacology have led to improvements in the cognitive performance of people with neurological disease and other forms of cognitive decline. These same methods may also afford cognitive enhancement in people of otherwise normal cognitive abilities. “Cosmetic”, or supranormal, cognitive enhancement offers opportunities to enrich our social or financial status, our interactions with others, and the common wealth of our community. It is common to focus on the potential benefits of cognitive enhancement, while being less than clear about the possible drawbacks. Here I examine the harms or side-effects associated with a range of cognitive enhancement interventions. I propose a taxonomy of harms in cognitive enhancement, with harms classified as (neuro)biological, ethical, or societal. Biological harms are those that directly affect the person’s biological functioning, such as when a drug affects a person’s mood or autonomic function. Ethical harms are those that touch on issues such as fairness and cheating, or on erosion of autonomy and coercion. Societal harms are harms that affect whole populations, and which are normally the province of governments, such as the use of enhancement in military contexts. This taxonomy of harms will help to focus the debate around the use and regulation of cognitive enhancement. In particular it will help to clarify the appropriate network of stakeholders who should take an interest in each potential harm, and in minimizing the impact of these harms.

## Introduction

Practical ethical decisions about cognitive enhancement require an analysis of the potential benefits and the potential harms of any drug or technology. But “harm” rarely means the same thing in different contexts or when used by different people. So there is a need for greater clarity in debates where potential harms and potential benefits are being balanced. Here I will outline one possible way to organize our thinking around the harms of cognitive enhancement, with the aims of clarifying the types of harms that may be encountered, and of understanding which groups may take an interest in each type of harm.

In this review article I propose that the harms that we encounter in cognitive enhancement fall into three categories:

I. (Neuro)biological harms
II. Ethical harms
III. Societal harms.

I will examine each of these categories, and show how a framework such as this helps in guiding thinking about the desirability of particular routes to cognitive enhancement. Although not intended as a hierarchy or scale of seriousness, these harms are roughly grouped in order of their directness, or immediacy from application of the enhancer.

To focus the discussion, this review article will only consider pharmacological enhancers and device-based enhancements. What does this focus exclude? Primarily it excludes lifestyle-based enhancement techniques such as getting plenty of sleep, avoiding recreational drugs and alcohol, and having a healthy diet and getting plenty of exercise. It excludes paying attention in school and reading for pleasure. These are proven, efficacious techniques that are not usually ethically troubling. It also excludes cognitive training and training via neurofeedback, which are techniques of uncertain efficacy and do not have the quasi-invasive nature of drug- or device-based techniques (Maslen et al., 2014).

Pharmacological methods involve the ingestion of pills or potions that cross the blood-brain barrier to affect the activity of the brain. Different drugs affect the brain in different ways. For example, the common putative enhancing drug methylphenidate (Ritalin) works to increase levels of dopamine in the brain by increasing dopamine availability through an effect on its receptors (Volkow et al., 2001). By contrast, mood-enhancing drugs such as Prozac act by slowing the rate at which the neurotransmitter serotonin is re-absorbed by nerve cells, so making more serotonin available in the circuits that control positive mood (Nutt et al., 1999).

Several qualitatively different types of cognitive enhancement device are available. Devices that use externally-applied electrical or magnetic energy to alter the excitability of brain function have been in use in neuroscience labs and clinics for some decades. Of these, much interest surrounds transcranial electrical stimulation (tES), and particularly the form that uses direct current (tDCS), since the devices are already appearing on the consumer market (Maslen et al., 2014; Wexler, 2015), and are relatively cheap and easy to manufacture at home (Fitz and Reiner, 2015). Neuroscientists frequently refer to transcranial magnetic or electrical stimulation as “non-invasive brain stimulation” or NIBS (Davis and van Koningsbruggen, 2013). Deep-brain stimulation (DBS) involves surgical implantation of electrodes into a target brain structure to deliver electrical pulses that enhance or disrupt neural transmission. The impracticality of access to DBS means that it is beyond the scope of this discussion, but it is worth noting that the ability of DBS to target deep neural structures and pathways that underlie reward and motivational behavior make it a technique for possible future concern (Synofzik and Schlaepfer, 2008).

## The Need for a Taxonomy of Harms

The notion of harm in cognitive enhancement is rarely dealt with in any detail. Neuroscientific publications may discuss the potential direct, physical harm that a person may experience from the enhancer or procedure, such as the risk of seizure, but usually framed in the context of criteria that exclude groups of people from a lab-based experiment. At the other extreme, ethical discussions of cognitive enhancement may propose a hypothetical, side-effect free enhancer, the use of which addresses a philosophical question of fairness or obligation, such as whether a surgeon has a duty to take a pill that keeps her alert. It is rare to find a discussion of specific harms that arise from a specific technique, and which may concern a specific group of people. Here I will attempt to give a framework for understanding different forms of harm, and give specific examples of where these harms may arise from procedures that are in common use.

Taxonomising harms has two effects. First, we can see the type of harm generated by a specific enhancer. Second, we can identify the people or groups whose interests are affected by the enhancer. For example, a drug that has no known side-effects but which can be taken, undetected, to enhance performance in an examination would be of interest to a philosopher studying fairness, but would be of little interest to the medical community. Or a device with potentially serious side-effects, but the ability to enhance mind-reading during a military interrogation, may be a matter for international treaties.

In the following sections I will deal with each of the three classes of harms of this taxonomy. I will discuss some examples of harms within each category, and I will summarize each class with an indication of who are the stakeholders in any discussion of those harms, and how discussions around these harms might be shaped.

## I. (Neuro)Biological Harms

In this category are harms that arise directly from the enhancer itself. Many of the harms in this category might normally be called *side-effects*, although I also include here effects that go beyond the “usual” list of side-effects that one commonly encounters. Most, but not all, of these harms have their effect on the brain or the central nervous system, but it would be more accurate to describe these as harms to the biological functioning of the person. Harms are listed here in terms of the biological response, although many harms may have common causes.

### Seizure

A seizure is an abnormal synchronization of electrical activity in the brain. Although many different factors may lead to a seizure in brain tissue, it is of particular concern in cognitive enhancement since pharmacological or device-based enhancers often work by changing cortical excitability. Artificial modulation of brain excitability is always treated with caution in a professional setting. For example, most research institutions exclude people from experiments with NIBS if they show elevated risk factors for seizure, including family history of epilepsy, or recent use of drugs or alcohol (Davis et al., 2013). Seizure is a rare complication in professional settings, with only a single seizure reported after tDCS (Ekici, 2015) and an estimated risk of less than 4% even in people who already have epilepsy (Schrader et al., 2004). However although the risk may be small for any individual person or procedure, the incidence of serious adverse effects is likely to increase as the use of enhancers becomes more widespread.

### Mood Changes

Many modern uses of neurointervention involve treatments for mood disorders. Applying tDCS across the prefrontal cortex over several sessions has been shown to lift mood in non-depressed and in depressed individuals (Brunoni et al., 2011b; Austin et al., 2016). However the electrode positions used in these studies (the F3/F4 positions of the International 10-10 system) are also used as targets in several studies of cognitive and memory function. So the possibility exists that interventions for enhancement of memory may lead to unintended changes in mood.

### Drug Interactions

When a drug such as methylphenidate is prescribed by a physician to treat a deficit, it will usually be accompanied by a list of other drugs that may or should not be taken at the same time. For example, it may be recommended not to drink alcohol or take sleeping pills. When enhancers are taken outside of this controlled environment, such as for cognitive enhancement, it is less clear how prescription or controlled drugs may interact with the enhancer. Enhancers may be taken at higher doses than usual or in combination with stimulants that may risk autonomic complications, as is already being seen on college campuses in the US (McCabe et al., 2005). Much less is known about how NIBS may be affected by other drugs. The long-lasting effects of NIBS seem to rely on a range of neurotransmitters, including dopamine, N-methyl-D-aspartate (NMDA) and serotonin (Filmer et al., 2014). For example, blocking NMDA receptors abolishes the long-lasting benefits of stimulation (Nitsche et al., 2003), so any psychoactive medication that operates via these mechanisms will interact with any enhancer that also relies on these same substrates. What could be harmful in not knowing these interaction effects? First, a user who is not satisfied with the effect of an enhancer (due to its being blocked by another drug) may increase their dose of the enhancer, placing them at greater risk of the side-effects of the enhancing drug or device. Second, drugs and devices may interact with each other in unknown ways, manifesting as effects on the central nervous system, or on the other vital systems of the body. As enhancers become more widely used, it will be increasingly important to communicate these risks to at-home users.

### Skin Lesions

Applying energy across the scalp means a build-up of electrical charge at the boundary between tissues of differing electrical properties. This is the source of the effect of tDCS, where modeling studies suggest that charge accumulates at the boundary between the highly conductive cerebrospinal fluid and the highly insulating gray matter of the cortex (e.g., Sadleir et al., 2010). However the boundary between the electrode and the scalp is another area where charge may accumulate, and this accumulation may be experienced by the participant as a warm or hot sensation. Poorly applied electrodes could burn the scalp, although in practice the electrodes are readily removed so this rarely happens in controlled settings (Brunoni et al., 2011a). Factors that seem to increase the risk of skin reactions, including irritation, burns or contact dermatitis, include long duration protocols with high current, and the salinity of the solution used to soak the electrodes (Bikson et al., 2016; Matsumoto and Ugawa, 2017). The long-term effects of chronic electrode use are not known.

### Addiction and Dependence

People may become dependent on stimuli that activate their reward systems. For example, nicotine is a highly addictive compound that appears to increase dopamine availability in the midbrain (Rice and Cragg, 2004) so activating reward pathways, and opiates such as heroin act directly on opiate-mediated reward pathways (Koob, 1992). Could a person become addicted to or dependent on cognitive enhancement? Ritalin and Modafinil are not thought to be addictive in their own right, and have even been suggested as a means of enhancing treatment for addiction to other drugs (e.g., cocaine: Grabowski et al., 1997; Martínez-Raga et al., 2008). Rats with electrodes implanted in midbrain reward structures are known to show signs of addictive behavior such as frequent self-administration at the expense of eating or drinking (Koob, 1992), although the homologous brain areas in humans would be accessible only to DBS and so are beyond this discussion. However human addiction is a multi-component process that includes many cortex-mediated functions such as cue association, behavioral control and mood monitoring (George and Koob, 2010). It would therefore seem possible that a person could trigger addictive-cycle behavior as an unintended consequence of manipulating involved functions. For example, in one study tDCS of prefrontal cortex to enhance memory (Berryhill and Jones, 2012) used the same protocol as another study that lifted mood (Austin et al., 2016); so one consequence of memory enhancement might be an unintended association of the procedure with a boost in happiness, setting up a reward cycle. There is also the possibility that people may become dependent in a less direct sense, through the development of a belief that enhancement is necessary in maintaining their current productivity or status; this point is developed later (*shifting norms*, below).

### Zero-Sum Trade-Offs

Scientific explorations of enhancement using NIBS typically set out to discover if a particular method has an effect on a particular faculty. It is regrettably rare for experimental work to look for off-target effects (Davis et al., 2013). This is despite two well-known features of brain stimulation: first that the energy delivered to the brain during stimulation is never isolated to the target region of the brain, and second that no brain area concerns itself only with a single function. So the likelihood of adverse effects in a function other than the one under scrutiny varies from *possible* in primary motor or sensory areas to *unavoidable* in some highly-connected areas such as the prefrontal cortex, as suggested above under *mood changes*. Certainly the rare works that test for off-target effects do show trade-offs such as enhanced learning at the cost of automaticity and *vice versa* (Iuculano and Cohen Kadosh, 2013). Some authors have gone as far as to suggest that such trade-offs are an inevitable consequence of the function of the brain (Brem et al., 2014), although the evidence for this position is rather scant (Luber, 2014). The picture is complicated further by the finding that even within a given protocol, individuals respond very differently; even for stimulation of the primary motor cortex, the “gold standard” of protocols, there is wide variability in response (Wassermann, 2002; Wiethoff et al., 2014). Other studies have shown paradoxical excitation with supposedly inhibitory stimulation (Batsikadze et al., 2013), or qualitatively different responses between younger and older participants (Heise et al., 2014). These findings highlight the need to examine individual differences, as well as presenting group results, in understanding enhancement protocols.

### Summary

In this section I have discussed some of the possible harms that a person may experience as a direct result of taking an enhancer. Harms could be clustered according to how the enhancer creates the harmful effect: so changes in mood and changes in seizure threshold both arise from the changes in cortical excitability induced by the enhancer. Alternatively, one could divide these harms into short-lived or long-lasting harms: a skin lesion may last for hours or maybe a day or two, while an alteration in emotional processing may last for months or years (which is desirable, in the case of depression). However we classify them, these harms might properly be called *side-effects*, and are equivalent to the side-effects listed on the packaging of pharmaceuticals. Determining the likely (neuro)biological effect of an enhancer is the responsibility of the manufacturer and of the person delivering the enhancer to another person. So in a university laboratory the researcher may rely on the manufacturer of an enhancing device to have adhered to proper engineering standards, and must take responsibility for correct application of the device to a participant and for following accepted safety guidelines. It is also the researcher’s duty to monitor for adverse effects of the enhancer, and to ensure the comfort and safety of the participant at all times. In a wider sense, it is the researcher’s responsibility to contribute knowledge of these harms to the community through good scientific practice, namely: conducting well-designed and well-controlled experiments; reporting the results of all experiments, including ones with null or “inconvenient” results; and reporting any adverse effect resulting from the experiment.

## II. Ethical Harms

Ethical harms here are those which do not arise directly from the specific enhancer, but rather from the covert or non-reciprocal use of an enhancer, or from the use of an enhancer in new contexts. Bioethical analysis almost invariably considers interactions among a small number of people, where one person may act unfairly towards another, or may seek to gain an advantage within a group. The recent rise of cognitive enhancement methods has generated much interest among philosophers, as a novel angle on the longer debate around enhancing physical capabilities.

### Cheating

Covert use of an enhancer is always likely to be ethically problematic, compared to open use. Many discussions of pharmacological enhancement focus on the use of drugs such as Modafinil on college campuses, where students may gain (perceived) advantages in examinations or coursework through the use of drugs designed to treat neurological conditions. Covert use of enhancement in education is invariably taken to be wrong in such discussions. Analogously, enhancement of sporting performance (“doping”) may be perceived as allowable or not according to one’s view of what is the goal of sport (Savulescu et al., 2004). However one views enhancement in sport, if the rules deem an intervention to be wrong, then using the intervention is by definition cheating and covert use of the intervention is particularly wrong. The seeming success of NIBS in enhancing cognitive and motor performance in laboratory settings means that it is inevitable that we should start to see neurointerventions in sports (“neurodoping”: Davis, 2013). However, just as in sports doping, the pressure to take enhancing substances leads people to take potentially harmful substances in order to keep up with their peers (Sjöqvist et al., 2008; Forlini and Racine, 2009). In many cases this pressure may come from a person’s organization, which raises questions about coercion (Dryden, 2006).

### Authenticity and Naturalness

A common worry about enhancement is that it diminishes and devalues the authenticity of our achievements. Again the model of drug enhancement in sport is instructive—we are disappointed when we learn that an athlete has been stripped of his medals after failing a drug test. Should we feel the same when a person excels in an examination, but has done so having taken Modafinil? And what should that person think about their own achievement? It could be that an outcome achieved in the context of enhancement is in some way less valuable than one made without, as it has removed the person from their authentic desires and abilities. A counter argument might be that if a person truly has a desire to achieve the highest possible score in a test and truly does not feel that enhancement is wrong, then that achievement is authentic for the person. There is no simple definition of authenticity, and debates on authenticity in enhancement can reveal more about the debater’s preferences for human flourishing than clarity on the acceptability of the practice. Nevertheless there does seem to be some inertia on the part of the public to accept cognitive enhancement methods as described here, possibly resulting from the perceived unnaturalness of the enhancer compared to methods such as exercise and good sleep (Caviola and Faber, 2015). A possible reason might be the view of Santoni de Sio et al. (2016) that taking an exam after taking an enhancer means that the person is effectively playing a different game—the “game” in an exam is to test a person’s natural ability to retain and recall information, so changing the way in which information is learned makes the game into one of expanding capacity. Santoni de Sio et al. (2016) distinguish between activities where the goal is to produce a certain outcome (goal-oriented activities, such as producing a rock album) and activities where the process itself is tested (practice-oriented activities, such as marathon running). Some work is required here to clarify issues around praiseworthiness for enhanced acts, and to determine when one person’s enhancement is beneficial or detrimental to the activity as a whole.

### Unequal Access

Justice, or equal treatment for all, is a core principle of bioethical analysis. Is there a risk of unequal access to enhancers? At present it is easy and cheap to buy putative enhancement drugs such as Modafinil from the internet (Schepis et al., 2008), and tDCS devices can be bought cheaply or even made at home for minimal cost (Jwa, 2015). So there seems to be no particular barrier to access to enhancers among people with access to the internet and who have sufficient cash to invest in them. Naturally this position favors the middle classes, who have the resources and the awareness to access enhancing products, but such disfavoring of people on lower incomes is not unique to the sphere of cognitive enhancement.

### Enhancement in Children

Any ethical discussion is necessarily amplified when applied to children. Most (but not all: Lebel et al., 2008) of the brain’s development occurs during the period of life before the person is considered to have responsible independance, yet is also the time when educational assessment often sets up a child’s future earning potential. There is therefore pressure amongst parents and peers to use any fair means possible to perform better in examinations, or to gather a portfolio of achievements in extra-curricular activities. The particular ethical worries here are around the ability of a child to make informed decisions about her own cognitive enhancement, and about the reliability of our knowledge of the effects of different enhancers on the child. In the former situation many of the arguments are shared with other biomedical procedures, where a child is required to assent to a procedure, but only a legally responsible person such as a parent can give consent. The latter situation reveals a gap in our understanding of the effects of many enhancers, particularly NIBS, and our inappropriate tendency to treat children as “small adults” when setting dosage (Davis, 2014). The ethical pressure in enhancing children is intensified when considering that any effects of enhancement, good or ill, are likely to affect the brain’s development for the rest of the child’s life, since the brain is in a more plastic state during the early years (Lenn, 1991). Given this, it is important that we consider carefully the arguments that may be used to justify cognitive enhancement in children (Krutzinna, 2016).

### Third-Party Risk and Responsibility

Much ethical analysis of cognitive enhancement focuses on the immediate rightness or wrongness of enhancing a particular action. However many actions also, at least potentially, involve a wider group of people than those immediately involved in the enhancement. It is clear that enhancing one person’s performance in a school test may cost another person their place in the ranking table. But the harms may extend beyond the test. We saw above that tDCS has been shown to enhance learning, but at the cost of automaticity (Iuculano and Cohen Kadosh, 2013). A recent study showed that tDCS could be used to aid flight training in pilots (Choe et al., 2016), however an important question remains whether the accelerated learning during the tDCS-enhanced training phase may come at the cost of impaired reactivity during real-world piloting. Given that commercial airline pilots may be responsible for transporting several hundred people at a time, it would be important to monitor the progress of training-enhanced pilots if the technology proliferates.

### Summary

The above examples of ethical harms reveal situations where cognitive enhancement may threaten accepted practices, or introduce sources of unfairness between people. Although there are active theoretical discussions of the ethics of enhancement (e.g., Savulescu and Bostrom, 2009), this section has looked at the practical ethics of determining potential harms in individual cases. Without a solid set of guiding principles for enhancement, it is a matter for individual regulators to determine what is fair or right, or how to incorporate enhancement into their regulated practices. So the responsibility for examining the harms lies with bodies that oversee the domain of interest. In the educational setting it is likely that a school or university will take a stand on the permissibility of using enhancers, and this may be a matter for a federation of institutions to produce collective guidelines. Where a third party may be harmed as a result of a person’s enhancement, it may require clarification or modification of the law. This process requires input from the users of enhancement drugs or devices to understand their needs, and from manufacturers and researchers who can advise on the safety and capability of the enhancers. It would then be a matter for the overseeing body to relate these needs and limitations to the values of the activity, to produce guidelines for the use of enhancers, and to monitor and enforce these guidelines.

## III. Societal Harms

The preceding section discussed how cognitive enhancement may impinge on interactions between people, or on the rightness of enhancing certain actions. However the widening access to enhancers will make these ethical questions more prevalent and more pressing. This means that we will need to address the societal implications of widespread access to enhancement. It is at this level that moral concerns become more pressing, where “morals” are taken to be the prevailing values or codes of conduct of a particular society. While there is no particularly satisfactory definition of moral conduct, nor an agreement about where morals may come from, there is at least a sense that we know when an agent acts immorally. In this section I will consider potential harms to society at large when cognitive enhancement becomes more widespread or extends to areas where moral conduct is crucial.

### Shifting Norms

One possible outcome of limited use of cognitive enhancement is that the restrictions upon their use become relaxed or eroded, and the default state of society moves from one where enhancement is exceptional to a state where to refuse enhancement would be considered remarkable. There are two ways in which the default, or norm, might shift. In the first case there may be a perceived advantage in taking an enhancer, with the advantage being sufficient to outweigh any possible harms. In this case an “arms race” of overt or covert consumption could develop where no individual wants to be left behind. In the second case the norm drifts more passively, that is without the pressure of competition, but with the caveat that once a person starts using an enhancer it would be seen as perverse to give it up. This pattern has already begun to emerge in the context of academic cognitive enhancement (Forlini and Racine, 2009). These cases are not mutually exclusive: for example, the use of “chalk” (magnesium carbonate) proliferated among rock climbers during the 1980s as a way to dry sweat from the fingertips. There were several prominent ethical objections to the use of chalk, namely that it caused environmental damage, that it made it easier for subsequent climbers to follow a line of white chalk-marked holds on the rock, and that the use of chalk as an enhancer devalued the achievement of a difficult ascent. Despite these objections, the use of chalk spread both through its use in competitive elite climbing and by hobbyist climbers, and is now so common in most European and North American climbing areas that it is easy to forget that there is little evidence that it confers any real benefit (Li et al., 2001). Thus the use of chalk may serve as a historical model for how the use of an enhancer of minimal real benefit may spread within a community through different mechanisms, until its use becomes the norm.

### Respect for Human Variation

People vary in all aspects of their personhood. We differ in our taste in music, in our haircuts and in our favorite childhood cartoon character. However we also vary in how fast we can run, in how much money we earn and how skilled we are at concealing wrong behavior. All of these latter attributes are potential targets for enhancement. While it may be desirable (at least to an individual) to change their potential to learn information, we risk a lack of respect for those who do not or cannot make the same changes, and a lack of respect for the notion that the variation among people is exactly what defines our individuality (Jordan, 1921). It is considered socially unpleasant when a person looks down on another for having less money, and it is also unpleasant (or, should be) when a person looks down on another for being less intelligent. Widening access to cognitive enhancement will make this social inequality more pressing for those who do not enhance. Associated with respect for human variation is respect for disability. Many conditions of disability are associated with qualitative differences in brain structure, that make it practically or absolutely impossible to raise cognitive capacities to even to the average level of the unaffected population. Although bioethicists would generally consider this to be *treatment* rather than *enhancement*, and therefore relatively unproblematic (e.g., Schwartz, 2005), it reflects a dimension on which people may vary, and where there may be pressure to change.

### Military and Security Uses

The defense industry has a long-standing interest in human enhancement, both for offensive and defensive purposes. Amphetamines have a long history of use in the military, for their ability to promote wakefulness and alertness during long operations. The US Air Force has a programme that generates enormously useful declassified information on the limits and the safety of tDCS in particular (see the Air Force Research Laboratory at Wright-Patterson Air Force Base)^1^. The recent rise of enhancement technologies has led to concerns about their possible use in a military context. At heart is the erosion of the moral responsibility of military personnel, who have a duty to obey the accepted rules of military engagement. Moral responsibility of personnel during warfare is a complex equation involving many factors, including those of legal obligations and of the duty to obey a superior’s orders. There has long been concern that enhancers such as amphetamines may diminish the capability and the responsibility of an agent during action (e.g., Bower and Phelan, 2003). Wolfendale (2008) discusses the moral difficulties of technologies that dissociate an agent from his or her actions, and the importance of centering moral responsibility on the agent. Moral disengagement is a feature of a person’s ability to commit heinous acts (Bandura, 1999), and many common pharmaceutical enhancers may affect a person’s moral judgement (Crockett, 2014). The secret nature of military research means that many of these potential societal harms may not come to light until it is too late for the usual deliberative process of academic thought to be more than a coroner’s inquest.

### Obligations and Duties

To this point we have considered the use of an enhancer as a voluntary decision, albeit one which may be more likely to be made if societal norms shift. But could there be situations where a person could be compelled to use an enhancer? For example, could a surgeon be compelled to take a pill that would keep her awake and alert for the duration of a long and complex surgery? Goold and Maslen (2014) suggest, on grounds of legal pragmatics and legal philosophy, that English law at least would not hold a surgeon liable for refusing an enhancer. The strong presumption of a person’s right to bodily integrity generally overrides the perceived advantages of compulsory medication, and Goold and Maslen (2014) note that the law treats negligence by omission less seriously than negligence by commission. At present, at least, it would seem that arguments of bodily integrity, or more precisely mental or cognitive integrity (Bublitz, 2013), would supervene over any attempt to enforce an obligation to enhance, and any variation of this right would likely require transnational action. However there may be cultural obligations to enhance that are less susceptible to legal oversight. For example, many workplaces are notorious for the degree to which employees are expected to work long hours or to conspicuously consume alcohol and other drugs in the name of workplace bonding or client management (Frone, 2003; Pidd et al., 2011). While regulators have made some efforts to address many illegal or antisocial practices, it is clear that junior employees feel significant pressure to conform to the local norms, and that such a coercive atmosphere requires a concerted, or possibly generational, change if it remains resistant to the general societal view of enhancement.

### Neurointerventions in the Criminal Legal System

The criminal justice system relies on establishing the true course of events, and on determining a level of punishment for wrongdoing. This process is inevitably imperfect, but there is an interest in methods that increase the reliability of judgements and the appropriateness of punishment. So far the law has been reluctant to use neuroscience as probative evidence, although neuroscience may be introduced in many jurisdictions. One controversial use of neuroscience is the possibility of uncovering concealed knowledge, either through prompting a confession from a suspect or from increasing the reliability of an eyewitness’s memory (Moreno, 2009). These avenues suffer from the problem that they add an additional layer of epistemological uncertainty onto a process that is already inexact (Rakoff, 2008). There is also the possibility that jurors and judges may be swayed by the so-called “seductive allure” of neuroscientific methods and imagery (Weisberg et al., 2008), although this worry may be unfounded (Farah and Hook, 2013; Roskies et al., 2013). It seems unlikely that neuroenhancement may be used in the near future in establishing guilt or motivation in criminal law. However there may be more use for enhancement in rehabilitating offenders. For example, Cabrera and Elger (2016) discuss the possibility of heightening or dampening the emotional component of offenders’ memories as an aid to promoting remorse or reducing the satisfaction gained from a crime. Alternatively the antecedents of crime such as addiction (Fecteau et al., 2010) or antisocial personality tendencies (Canavero, 2014) may be susceptible to neuromodulation, although other ethical barriers may intervene.

### Summary

Societal harms are harms that threaten the stability of societies. That is, they may threaten the stable values of a community, or threaten the stability of interactions between nations. This is not to say that societies are unable to absorb change. But such change usually requires acts of government or debates between nations. For example, the conduct of war is guided by treaties that establish reasonable practice, such as the use of chemical weapons or the treatment of prisoners. These treaties can be changed or added to (such as when nations change their position on the use of nuclear weapons), but this again requires concerted political and diplomatic effort. So the bodies responsible for understanding the effects of cognitive enhancement on society are those that represent the moral character of that society. Depending on the society, that may be the democratic representatives of the population, or the religious leaders, or senior legal experts, or non-governmental organizations that press for change. These bodies need input from researchers and primary users, and take direction from philosophers and legal scholars.

## Conclusions

In this review article I have attempted to set out a framework for assessing the harms arising from a particular enhancement intervention, and for identifying the interested parties whose expertise may be required in judging the relative acceptability of the enhancer given all the available information. For example, a neuroscientist considering using tDCS to enhance a cohort of undergraduates’ ability to memorise strings of numbers may need to know about the side-effects of tDCS, but may not be overly concerned with the potential use of tDCS in manipulating political beliefs. Conversely, a government’s defense minister will need to understand the societal implications of authorizing the use of tDCS to extract information from a battlefield prisoner, but may be less concerned with the risk of skin lesions in that prisoner.

This is not to suggest that a politician need be ignorant of the side-effects, nor that a scientist should close her ears to issues of military uses of enhancement technology. However the level at which a person considers the issues surrounding enhancement technologies is generally limited. The three-level taxonomy of harms that I propose here, of biological, ethical and societal harms, will help in guiding the discussion of cognitive enhancement for a particular enhancer or a particular use.

I have not dealt here with the issue of who, precisely, is best placed to make decisions around the use of cognitive enhancement in a given situation. There are many domains, such as professional sports, where there are many layers of regulatory oversight, and many different demands and obligations on the person taking the enhancer. For example, a drug may be banned by criminal law in an athlete’s home country but not the country of competition, or the athlete may have signed a civil contract with a sponsor not to take that drug, or the sport’s governing body may allow a drug in some circumstances but not others. Navigating overlapping systems is always a fraught process (Black, 2008; Yeung and Dixon-Woods, 2010). However if we are clear about the types of harms that may be encountered in cognitive enhancement, we can at least help these competing regimes to determine where they fit in the regulatory geography.

Good policy decisions in biomedicine can only arise through an interaction among legal experts, ethicists and scientists, with the goals of bringing greater reflective practice to ethically significant science, and of grounding speculative reasoning in scientific possibility (Maslen, 2015). I would also emphasize the need to engage stakeholders at all levels of this debate, including the end-users of the enhancement products (Davis, 2016), to understand each person’s expectations and responsibilities, and at the same time offering advice to users about potential risks and harms. In particular, I argue that greater and more thoughtful prominence must be given to the harms associated with each possible means of enhancement. Using a taxonomy such as the three-part system outlined here gives a structure to this debate, and clarifies the level of engagement of parties that take an interest in how cognitive enhancement is used. There is no doubt that drug- or device-based cognitive enhancement will increase in usage and in ingenuity. It is of benefit to all that all parties discuss the risks as well as the benefits of these methods.

## Author Contributions

NJD conceived and wrote this article.

## Conflict of Interest Statement

The author declares that the research was conducted in the absence of any commercial or financial relationships that could be construed as a potential conflict of interest.

## Footnotes

^1^http://www.wpafb.af.mil/afrl/711hpw
